# Insight Into Function and Subcellular Localization of *Plasmopara viticola* Putative RxLR Effectors

**DOI:** 10.3389/fmicb.2020.00692

**Published:** 2020-04-21

**Authors:** Tingting Chen, Ruiqi Liu, Mengru Dou, Mengyuan Li, Meijie Li, Xiao Yin, Guo-tian Liu, Yuejin Wang, Yan Xu

**Affiliations:** ^1^State Key Laboratory of Crop Stress Biology in Arid Areas, Northwest A&F University, Yangling, China; ^2^College of Horticulture, Northwest A&F University, Yangling, China; ^3^Key Laboratory of Horticultural Plant Biology and Germplasm Innovation in Northwest China, Ministry of Agriculture, Northwest A&F University, Yangling, China

**Keywords:** grapevine, *Plasmopara viticola*, RxLR effectors, subnuclear localization, biological activities

## Abstract

Grapevine downy mildew, caused by oomycete fungus *Plasmopara viticola*, is one of the most devastating diseases of grapes across the major production regions of the world. Although many putative effector molecules have been identified from this pathogen, the functions of the majority of these are still unknown. In this study, we analyzed the potential function of 26 *P. viticola* effectors from the highly virulent strain YL. Using transient expression in leaf cells of the tobacco *Nicotiana benthamiana*, we found that the majority of the effectors could suppress cell death triggered by BAX and INF1, while seven could induce cell death. The subcellular localization of effectors in *N. benthamiana* was consistent with their localization in cells of *Vitis vinifera*. Those effectors that localized to the nucleus (17/26) showed a variety of subnuclear localization. Ten of the effectors localized predominantly to the nucleolus, whereas the remaining seven localized to nucleoplasm. Interestingly, five of the effectors were strongly related in sequence and showed identical subcellular localization, but had different functions in *N. benthamiana* leaves and expression patterns in grapevine in response to *P. viticola*. This study highlights the potential functional diversity of *P. viticola* effectors.

## Introduction

Grapevine downy mildew, caused by the oomycete *Plasmopara viticola* (Berk. & M. A. Curtis) Berl. & De Toni, is one of the most important diseases negatively impacting grape production worldwide (Panstruga and Dodds, 2009). As a biotrophic oomycete, *P. viticola* attacks almost all green tissues of the plant. In the asexual life cycle, an encysting zoospore forms a germinative tube which penetrates the leaf through a stoma, producing haustoria to obtain nutrients from the host (Panstruga and Dodds, 2009; Dodds and Rathjen, 2010).

Pathogenic oomycetes secrete effector molecules into the plant to disturb plant innate immunity, facilitating penetration and colonization (Judelson and Blanco, 2005; Kamoun, 2006; Tyler et al., 2006; Birch et al., 2009; Bozkurt et al., 2012; King et al., 2014). These effectors are divided into two important classes, apoplastic and cytoplasmic effectors. Cytoplasmic effectors include proteins of the RxLR and Crinkler (CRN) families (Mestre et al., 2016). The RxLR protein effectors are the largest class of effectors and have been the most extensively studied (Kamoun, 2006; Stassen and Van den Ackerveken, 2011). These comprise an amino- (N-)terminal signal peptide, followed by an RxLR motif (Arg-x-Leu-Arg, where x represents an any amino acid), followed by an EER motif (Kamoun, 2006; Birch et al., 2008). The signal peptide directs the secretion of the effector from the fungus, whereas the RxLR-EER motif participates in the delivery of the effector to the host cell (Whisson et al., 2007; Dou et al., 2008; Grouffaud et al., 2009). An exact RxLR-EER sequence is not always required for translocation to the host cell (Dou et al., 2008; Tian et al., 2011; Chen et al., 2013; Ye et al., 2015).

The battle between host and pathogen is multi-layered. Pathogen-associated molecular patterns (PAMPs) can be detected by pattern recognition receptor (PRR) proteins in the host cell membrane in a general mechanism referred to as PAMP-triggered immunity (PTI) (Jones and Dangl, 2006; Dodds and Rathjen, 2010). However, pathogens often secrete effectors inside the host cell to interfere with PTI, which the plant may respond to by effector-triggered immunity (ETI) (Dangl and Jones, 2001). For example, INF1, an elicitin secreted by *Phytophthora infestans* that acts as a PAMP, is recognized by the receptor-like protein ELICITIN RESPONSE (ELR), which then associates with the 1-ASSOCIATED KINASE1/SOMATIC EMBRYOGENESIS RECEPTOR KINASE 3 (BAK1/SERK3) protein kinase to trigger cell death (Du et al., 2015). This defense-related hypersensitive response (HR) has similirities with programmed cell death in induced by the pro-apoptotic mouse protein BAX (BCL2-Associated X) (Lacomme and Santa Cruz, 1999). However, oomycetes have evolved effectors that suppress PTI and ETI. For example, CRN70 and Avr1k, the two effectors of *P. Infestan*, have been showed to suppress a resistance (R) protein 3/AVR3a (gene to gene model)-induced HR (Kelley et al., 2010; Rajput et al., 2014; Fawke et al., 2015).

With recent advances in grapevine genomics and genomics technologies, rapid progress has been made in understanding resistance mechanisms to *P. viticola*. Numerous putative effectors have been identified from *P. viticola* based on their early expression during the infection process (Riemann et al., 2002; Sebastian et al., 2010; Mestre et al., 2012). Utilizing a cDNA-AFLP approach, 96 *P. viticola* sequences were obtained from infected grapevine leaves (Polesani et al., 2008). Additionally, many *P. viticola* cDNA sequences have been cataloged from expressed sequence tags (ESTs) from infected plant tissues (As-sadi et al., 2011; Cabral et al., 2011; Mestre et al., 2012). Fifty-four ESTs encoding potential secreted hydrolytic enzymes and effectors were identified from germinated zoospores of *P. viticola* (As-sadi et al., 2011; Cabral et al., 2011; Mestre et al., 2012). Based on the presence of a secretory signal sequence and an RxLR or CRN motif, 51 RxLR effectors and 10 CRN effectors were identified from *P. viticola* (Yin et al., 2015). RNA-based sequencing has been used to identify RxLR/CRN genes that are differentially expressed upon infection of grapevine (Brilli et al., 2018). The genome of *P. viticola* has been sequenced and hundreds of effectors including RxLR and CRN have been identified (Dussert et al., 2016, 2018; Yin et al., 2017). In recent years, there has been rapid progress in understanding *P. viticola* RxLR effectors. For example, the effector PvRxLR28, which can suppress cell death caused by some cell death elicitors, exhibits a burst of expression 6 h after infection (Xiang et al., 2016). This contributes to the pathogenicity of this strain. In contrast, the effector PvRxLR16 significantly enhances resistance, and can trigger cell death by itself in *N. benthamiana* cells (Xiang et al., 2017). A total of 83 putative RxLR effectors were identified from *P. viticola* “JL-7-2”; three of these were localized to chloroplasts while one was localized in both chloroplast and mitochondria (Liu et al., 2018). Effectors generally need to enter the host cell to carry out their function. The late blight resistance protein R1 and effector AVR1 require nuclear localization to activate the immune response, and PvRxLR16 and AVH241 need to localize to the nuclear and plasma membrane to trigger cell death, respectively (Yu et al., 2012; Du et al., 2015a; Xiang et al., 2017).

In this paper, we carried out genomic and RNA-based sequencing of the *P. viticola* strain “YL” (Yin et al., 2017). Based on nucleotide sequence, we identified a group of putative *P. viticola* RxLR effectors. Bioformatic surveys have revealed that a set of 25 *P. viticola* RxLR effectors were predicted in the genomic of the *P. viticola* (unpublished). One RxLR effector (PvAVH54804) was digged out during the infection of *V. vinifera* RNA-seq data (Liu et al., 2019). Here, we studied the virulence function of 26 of the RxLR effectors by transient expression in *N. benthamiana* cells. We examined both their ability to suppress cell death induced by INF1 and BAX, and their subcellular localization, both in *N. benthamiana* and *V. vinifera*. This provides a solid foundation for advanced study of the role of these and other effectors in grapevine downy mildew.

## Materials and Methods

### Bioinformatics and Sequence Analysis

The sequence of the putative *P. viticola* RxLR effectors in this paper can be found in GenBank date library of National Center for Biotechnology Information (NCBI), and all accession numbers were listed in Supplementary Table S2. Signal peptide cleavage sites were predicted using the SignalP 4.1 server^1^, and nuclear localization signals were identified using cNLS Mapper^2^. Sequences were compared with three published genome sequences (Dussert et al., 2016; Yin et al., 2017; Brilli et al., 2018). The phylogenetic tree was constructed with amino acid sequences using MEGA5 with the neighbor-joining method, 1,000 replicates, and the pairwise deletion option. Multiple alignment was carried out using BioEdit 7 software with ClustalW. Protein sequence alignment was performed using DNAMAN software.

### Bacterial Strains and Plasmid Constructions

*Escherichia coli* strain Top10 (CWBIO) was used for cloning and amplification of recombinant plasmids, and was cultured at 37°C in LB (Luria-Bertani) medium. *Agrobacterium tumefaciens* strain GV3101 was used for transient expression *in planta* and was grown at 28°C in LB medium. The open reading frame (ORF) segments of putative *P. viticola* effectors, minus the predicted N-terminal signal sequence, were cloned from genomic DNA of *P. viticola* strain “YL” by PCR amplification and specific primers designed based on genomic sequence. PCR products were cloned into the plant expression PVX vector and pCAMBIA2300 vector. For PVX assay, the amplified segments were ligated into PVX vector pGR107 (containing a HA tag sequence) to form PVX-effectors construction. To identify the subcellular localization of those effectors in plant, effector gene sequences were ligated into pCAMBIA2300 vector containing green fluorescent protein (GFP) sequence. The primers in this paper were listed in Supplementary Table S1. The schematic diagrams of the constructs used for experiments were documented in Supplementary Material, Supplementary Figure S8.

### Plant Materials, Transient Transformation Expression Assays

Recombinant plasmids were introduced into *Agrobacterium* by liquid nitrogen flash freezing, and transformants were selected on LB medium with kanamycin (50 μg/mL), gentamycin (50 μg/mL), and rifamycin (50 μg/mL). *Agrobacterium* containing effector-GFP constructs was cultured in liquid LB medium at 28°C with shaking at 200 rpm/min. After 20 h, cells were collected by centrifugation at 5,000 × *g* for 3 min, washed twice in 10 mM MgCl_2_, and then resuspended in infiltration buffer (10 mM MgCl_2_, 10 mM MES, pH 5.7, 200 μM acetosyringone) to a final OD_600_ of 0.4. Then, the solution was incubated for 3 h in the dark prior to infiltration as described (Van der Hoorn et al., 2000). Leaves of 4 to 5-week-old *N. benthamiana* and *V. vinifera* cv. Thompson seedless were subjected to infiltration using a 1-mL needless syringe. The healthy and fully expanded leaves of 4 to 5-week-old *N. benthamiana* leaves were used for protoplast preparation. The plant materials were grown in a greenhouse under 16-h light/8-h dark photoperiods at 25°C (light) and 18°C (dark). *P. viticola* isolate “YL” was maintain in grapevine leaves, and subcultured every 7 days at 22°C. To analyze expression levels of putative RxLR effectors, detached leaves of the susceptible *V. vinifera* cultivar “Pinot Noir” were inoculated with 40 μL *P. viticola* “YL” sporangial suspension (5 × 10^4^ sporangia / mL) / water (as negative contral) on wet sterile filter papers, and then placed in growth champer at 22°C. Expression was evaluated by RT-PCR at 0 h, 6 h, 12 h, 24 h, 48 h, 72 h, 96 h, and 120 h after inoculation as described in Liu et al. (2019). These experiments were repeated at least three independent experimental repetitions with similar results.

### Confocal Microscopy and Image Analysis

For characterizing the subcellular localization of putative *P. viticola* effectors, transient expression assays were conducted using leaves of 4 to 5-week-old *N. benthamiana* and *V. vinifera* plants with GFP-effector fusion proteins. Images were captured by confocal microscopy (Germany, LECIA) 3 days after infiltration. For transient expression in protoplasts, healthy and fully expanded *N. benthamiana* leaves were used for protoplast preparation. Protoplasts were subjected to transformation using a polyethylene glycol (PEG)-based method (Yoo et al., 2007). To unambiguously mark the nucleus, we also engineered and introduced an expression plasmid (pBI221-mCherry) fusing the NLS from SV40 T large antigen (amino acid sequence PKKKRKV) to a red fluorescent protein. Effector-GFP fusion proteins and GFP, together with NLS-mCherry which was considered as nuclear-localized marker were co-expressed in protoplasts following polyethylene glycol (PEG)-mediated transformation of the protoplasts. Protoplasts were incubated for 20 h under weak lighting at 25°C. Fluorescence of GFP and mCherry was excited at 488 nm and 561 nm, respectively.

### Measurement of Ion Leakage

For measurement of ion leakage, five, 1-cm diameter leaf disks were incubated in 5 ml distilled water with gentle shaking (50 rpm/min) for 3 h at room temperature. Ion leakage was measured by a conductivity meter (DDS-307, LeiCi, ShangHai, China) as A. Total ion leakage was measured as B after incubation in a boiling water bath for 25 min. Results were expressed as percentage of total ion leakage A/B.

### Protein Extraction and Western Blotting

Recombinant proteins were extracted from *N. benthamiana* leaves 2 days after infiltration using protein extraction buffer [50 mM HEPES-KOH, pH 7.5, 150 mM KCl, 1 mM EDTA, 0.5% (v/v) Triton X-100, 1 mM dithiothreitol, and 1 × protease inhibitor cocktail (ROCHE)]. Tissue was suspended in 1 × loading buffer, boiled in a water bath for 5 min, and subjected to centrifugation at 10,000 × *g* for 10 min. The supernatant containing solubilized protein was resolved on a 10% SDS-PAGE gel and transferred to a PVDF membrane. The membrane was blocked with 5% nonfat dry milk in TBST buffer (20 mM Tris–HCl, pH 8.0, 150 mM NaCl, 0.05% Tween-20) for 3 h. Mouse anti-GFP monoclonal antibody and anti-HA-monoclonal antibody (ABclonal) were added to the buffer at a 1:5000 dilution, and the membrane was shaken slowly at 4°C overnight. The membrane was then washed three times in TBST buffer, and goat anti-mouse IRDye 800CW in TBST was then added to the membrane at a dilution of 1:10000, and the membrane was shaken for an additional 1–2 h. The membrane was then washed 3–5 times for 5 min each in TBST and visualized using ChemiDoc^TM^ XRS+ Software with excitation at 700 and 800 nm.

### RNA Extraction and Expression Pattern Analysis

For analysis of gene expression following *P. viticola* infection, RNA was purified from inoculated leaves using the Plant RNA Kit (OMEGA, United States). The quality of total RNA was assessed by electrophoresis on 1% agarose gels, and RNA concentration was determined by spectrophotometer (NanoPhotometer^®^; IMPLEN, CA, United States). Total RNA was treated with gDNA wiper mix (Vazyme) before complementary DNA (cDNA) synthesis. Total RNA (500 ng) was reverse transcribed to cDNA in 20 μL volume using the HiScript^®^ Reverse Transcriptase Kit (Vazyme) according to instructions of the manufacturer. Oligonucleotide primers for RT-PCR assays were designed for specificity to each gene based on sequence information (Supplementary Table S2). A *PvActin* gene was used as an internal reference (Mestre et al., 2012).

## Results

### Identification and Phylogenetic Analysis of RxLR Effectors From *P. viticola* ‘YL’

**FIGURE 1:**
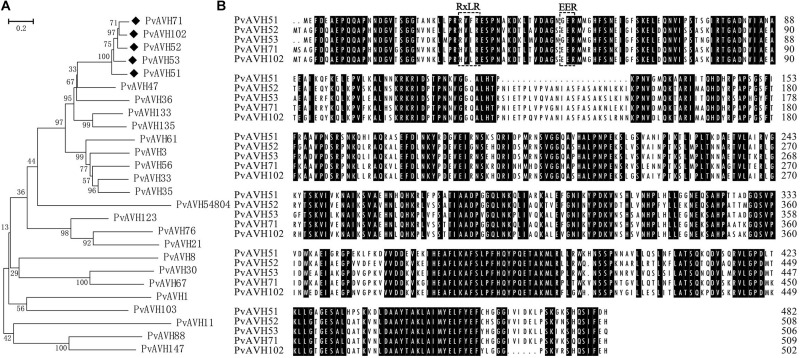
Phylogeneticrelationship of 26 putative RxLR effectors from *Plasmopara viticola*. **(A)** A phylogenetic tree was constructed from alignment of predicted amino acid sequences using MEGA 5 with the neighbor-joining method, 1,000 replicates, and the pairwise deletion option. Five very closely related proteins are indicated with black diamonds. **(B)** Amino acid sequence alignment of the five very closely related RxLR proteins (PvAVH51, 52, 53, 71, and 102). The RxLR and EER motifs are indicated with black boxes. Positions where the amino acid is conserved in all five proteins is shown with black background.

Based on the presence of an N-terminal, signal-like sequence of 17–30 amino acids and conserved RxLR and EER motif, we identified ∼100 putative RxLR effector genes from genomic sequence of the *P. viticola* isolate “YL.” Of these, 26 were selected for analyses of function and subcellular localization (Table 1 and Supplementary Table S3). Compared with the *P. viticola* genome data, 3, 4, and 6 effectors were indentified the same as the published genomes from *P. viticola* isolate “JL-7-2,” “INRA-PV221” and “PvitFEM01,” respectively. In addition, most of these genes had the same length, and sequences query cover reached up to 96% (25/26), 100% (26/26), and 69% (18/26), but there were 8, 3, and 7 effectors had gaps compared with the three genomes, respectively (Supplementary Tables S4–S6). Two of these, PvAVH8 and PvAVH103, were previously reported as PvRxLR11 and PvRxLR9, respectively (Xiang et al., 2016). The RxLR-EER motifs were located in the N-terminal 20–80 amino acids of the putative effectors. All candidate effectors contained both RxLR and EER motifs except for PvAVH33, which had only an RxLR motif. The deduced amino acid sequences were aligned for 26 proteins, and a phylogenetic tree was constructed (Figure 1). This revealed that five proteins (PvAVH51, 52, 53, 71, and 102) had an especially close relationship, with up to ∼88% sequence homology (Figures 1A,B). The corresponding genes for these five were located on the same chromosome. Because the signal peptide is cleaved upon secretion and not present in the mature protein (Bozkurt et al., 2012; Fawke et al., 2015; Gascuel et al., 2016), we cloned the ORFs, minus the signal peptide sequence, from *P. viticola* genomic DNA for further experiments.

**TABLE 1:** Summary of 26 putative *Plasmopara viticola* RxLR effectors description.

**Effectors**	**Peptide size**	**Signaip prediction**	**SignalP3-HMM**	**RxLR conserved motifs**	**EER motif**	**Effectors conserved prediction**	**Genomic location**
PvAVH1	312	1–20	1	RLLR	DEER	46–61	scaffold_4:1041171-1042109
PvAVH33	559	1–21	0.999	RLLR		29–32	scaffold_140:87996-89675
PvAVH36	726	1–19	1	RVLP	DLKNKWAVHAGGEDR	30–52	scaffold_128:53655-55835
PvAVH147	377	1–25	0.977	RGLK	DEEAR	21–43	scaffold_59:284672-285805
PvAVH3	642	1–21	0.999	RFLR	DEER	20–50	scaffold_584:8728-10656
PvAVH8(PvRxLR11^a^)	202	1–23	0.992	RSLD	DEER	49–61	scaffold_4:1177443-1178051
PvAVH76	630	1–19	0.997	RDSR	DEER	31–49	scaffold_19:447692-449584
PvAVH21	620	1–23	0.905	RFLR	DEER	28–49	scaffold_19:695886-697748
PvAVH123	117	1–23	0.938	RFLQ	DEER	46–67	scaffold_19:781377-781730
PvAVH11	514	1–25	0.989	RSLQ	DSEER	53–70	scaffold_76:145128-146672
PvAVH133	724	1–21	1	RILR	DEER	30–51	scaffold_128:156764-158938
PvAVH135	722	1–23	0.999	RILR	DEER	28–49	scaffold_128:171504-173672
PvAVH67	414	1–19	0.998	RFLL	DESR	36–51	scaffold_143:19374-20618
PvAVH53	526	1–21	0.999	RVIR	DAGNGER	30–51	scaffold_128:17264-18844
PvAVH103(PvRxLR9^b^)	116	1–17	0.987	RILR	DGNVNREQER	49–64	scaffold_141:183645-183995
PvAVH30	445	1–19	1	KLLR	DNATNESR	34–41	scaffold_143:88038-89375
PvAVH56	572	1–22	0.999	RDPK	DLKLSAGNEER	39–50	scaffold_140:107427-109145
PvAVH35	720	1–23	1	RNLK	DEER	37–52	scaffold_140:56540-58702
PvAVH61	729	1–19	0.999	RVLR	DFTLSAGNEER	30–52	scaffold_140:44341-46530
PvAVH51	502	1–21	0.999	RVFR	DAGNGER	30–51	scaffold_128:4980-6488
PvAVH52	526	1–19	0.999	HVLR	DAGSEER	31–53	scaffold_128:92002-93582
PvAVH71	527	1–19	0.988	HVLR	DAGSEER	31–53	scaffold_128:45536-47119
PvAVH102	520	1–19	0.998	HVLR	DAGSEER	31–53	scaffold_128:32477-34039
PvAVH47	399	1–25	0.999	RILR	DEER	30–57	scaffold_128:247-1446
PvAVH88	486	1–30	0.995	RNLA	DDER	36–73	scaffold_59:287955-289415
PvAVH54804	249	1–21	0.995	RPLG	DDTNGEDR	53–70	

***^*ab*^**Xiang et al. (2016).*

### A Subset of *P. viticola* RxLR Effectors Can Trigger Cell Death When Expressed in *N. benthamiana*

**FIGURE 2:**
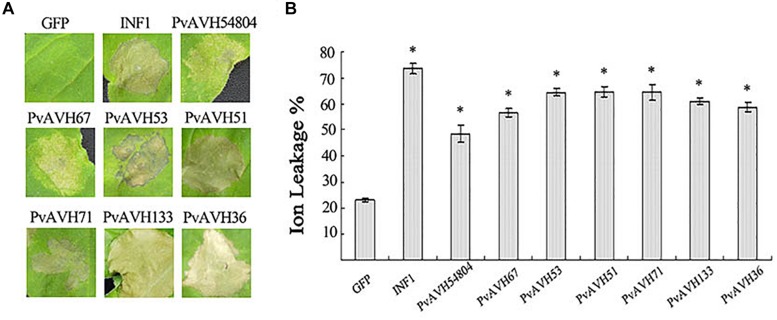
*P. viticola* effectors triggered cell death in *Nicotiana benthamiana*. **(A)** Photographs of *N. benthamiana* leaves subjected to infiltration with agrobacterium carrying PVX-effectors at 4 days post-infiltration. Leaves infiltrated with the empty vector control (GFP) or positive control (INF1) is shown as reference. The experiment was repeated at least three times; representative results are shown. **(B)** Electrolyte (ion) leakage from infiltrated tissue at 4 days post-infiltration. Values were determined from three independent repetitions. Error bars indicate SE, with asterisk indicating significant difference from the empty vector control (GFP). Data are means ± SE based on three independent replicates, each including 10 individual leaves. (^∗^ indicate *P* < 0.05 in one-way ANOVA, using Turky HSDa test).

As a first step to assess function of the 26 effectors, we expressed the ORFs in *N. benthamiana* leaf cells using *Agrobacterium* infiltration, and assessed their potential to induce cell death. The ORF sequences of the 26 PvRxLR effectors were ligated into the *PVX* vector and *pCAMBIA2300* vector as described in Yin et al. (2019). INF1, a known cell death inducer from *P. infestans*, and empty vector were used as positive and negative controls, respectively. We found that of the 26 effectors, expression of seven caused cell death by 4 days post infiltration (dpi) (Figure 2A). PvAVH51, 53, 71, 133, and 36 induced necrotic symptoms similar to those induced by INF1, although the effect of INF1 was more immediate (Figure 2A). Cells infiltrated with PvAVH54804 and PvAVH67 exhibited weaker cell death symptoms than the other five effectors. We also evaluated electrolyte leakage in the infiltrated *N. benthamiana* cells as an independent measure of cell death (Mittler et al., 1999). We found that ion leakage in leaves from plants transiently expressing the seven RxLR effectors was markedly higher than the empty vector control, but was less than the INF1-infiltrated plants (Figure 2B). These results showed that at least seven putative RxLR effectors from *P. viticola* could promote cell death when expressed heterologously in tobacco.

### Suppression of INF1- and BAX -Triggered Cell Death by *P. viticola* Effectors

**FIGURE 3:**
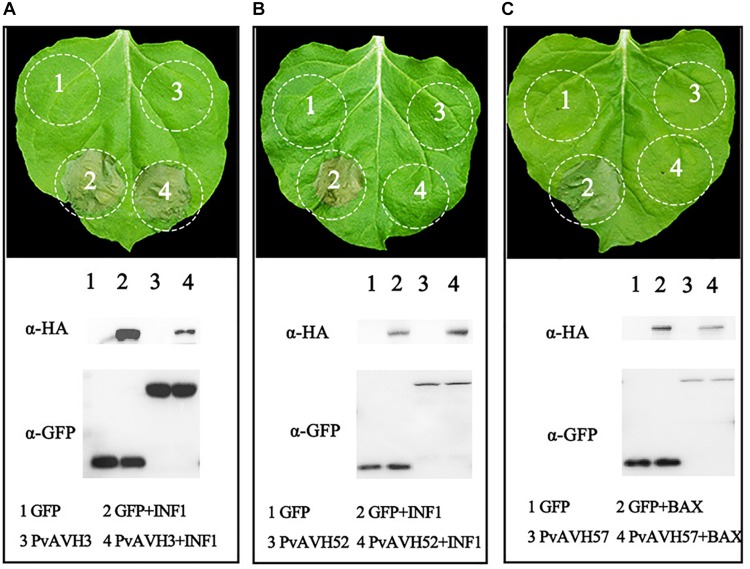
Suppression of PCD by *Plasmopara viticola* RxLR effectors. Three phenotypes observed upon expression of effectors in *N. benthamiana* are shown. Effector-GFP recombinant constructs and GFP control were transiently expressed on opposite sides of *N. benthamiana* leaves. **(A,B)** Different phenotypes: no suppression of INF1-triggered cell death (example, PvAVH3) and suppression of INF1-triggered cell death (example, PvAVH52); **(C)** All effectors tested could supress BAX-triggered cell death (example, PvAVH47). Proteins were extracted from infiltrated areas to analyze expression. Each infiltration results from more than three leaves; each infiltration test was repeated at three times with similar results.

To further analyze the function of the 26 putative *P. viticola* effectors, we evaluated their potential to suppress cell death induced by INF1 and BAX when co-infiltrated into *N. benthamiana* (Table 2 and Supplementary Figure S1), an approach previously used to explore effector function (Wang et al., 2011). *Agrobacterium* carrying the recombinant effector plasmid was infiltrated into *N. benthamiana* leaves 24 h after infiltration of BAX or INF1, and cell death was examined after a further 4 days. Seven effectors were identified induced cell death themselves in *N. benthamiana* in above, remaining 19 effectors were analyzed. The results showed that six putative RxLR effectors failed to suppress the activity of INF1 like PvAVH3, 13 effectors could suppress the INF1-induced cell death completly like PvAVH52 and 19 could suppress BAX like PvAVH 47 (Figures 3A–C and Supplementary Figure S1). Western blotting detected the proteins expression and showed the constructs successfully transformed in *N. benthamiana* leaves spots. Among these, thirteen could suppress both INF1- and BAX-triggered cell deaths (Supplementary Figure S1).

**TABLE 2:** Subcellular localization and suppression of cell deathinduced by BAX and INF1.

**Effectors**	**Subcellular localization^a^**	**Suppression of PCD^b^**		
		**INF1**	**BAX**	**PCD**
PvAVH1	cp	−	+	
PvAVH33	nc	+	+	
PvAVH36	n			cd
PvAVH147	nc	+	+	
PvAVH3	n	−	+	
PvAVH8(PvRxLR11)	nc	+	+	
PvAVH76	n	−	+	
PvAVH21	n	+	+	
PvAVH123	nc	+	+	
PvAVH11	cp	+	+	
PvAVH133	n			cd
PvAVH135	n	+	+	
PvAVH67	n			cd
PvAVH53	n			cd
PvAVH103(PvRxLR9)	n	+	+	
PvAVH30	n	+	+	
PvAVH56	nc	+	+	
PvAVH35	n	−	+	
PvAVH61	n	−	+	
PvAVH51	n			cd
PvAVH52	n	+	+	
PvAVH71	n			cd
PvAVH102	n	−	+	
PvAVH47	n	+	+	
PvAVH88	n	+	+	
PvAVH54804	nc			cd

*^*a*^ n, nuclear; nc, nuclear and cytoplasm; cp, cytoplasm and plasma membrane. ^*b*^ +, consistent suppression_;_ −, no suppression; cd, trigger cell death.*

### Subcellular Localization of 26 RxLR Effectors

**FIGURE 4:**
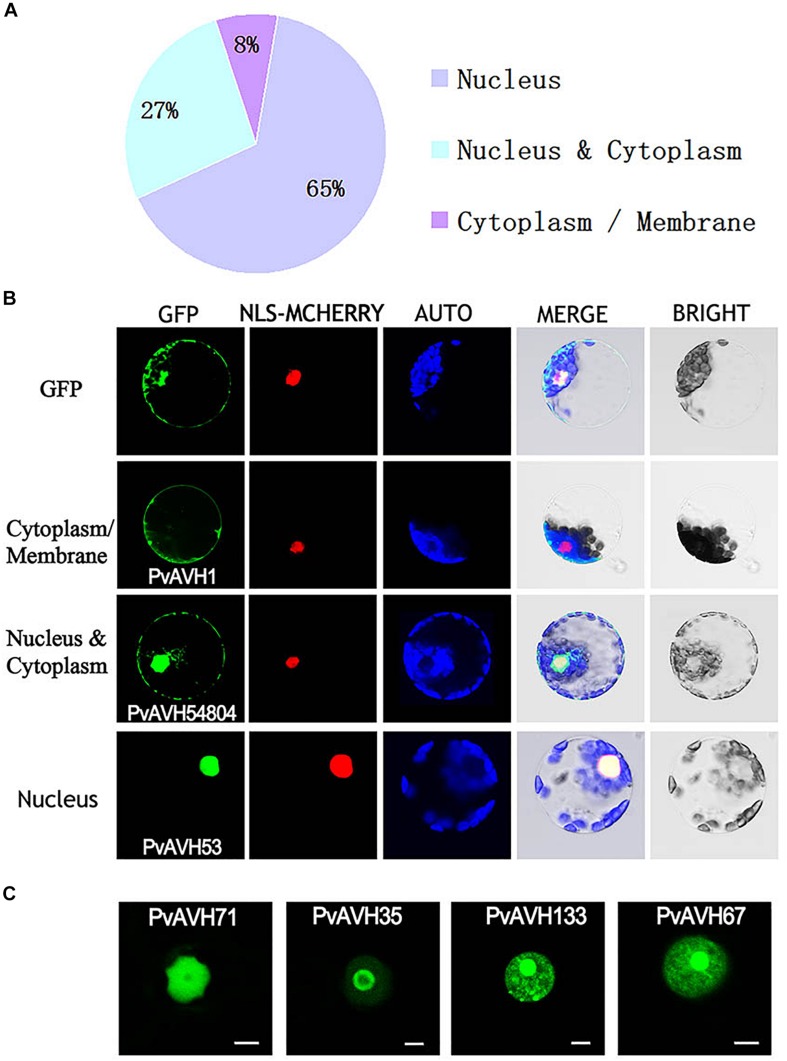
Subcellular localization of *Plasmopara viticola* RxLR effectors. Effector-GFP fusion proteins or GFP were expressed in *Nicotiana benthamiana*. Fluorescences accumulation of GFP and m-Cherry were analyzed by confocal microscopy, and excited at 488 nm and 561 nm, respectively. **(A)** Pie chart indicating the subcellular localization distribution for 26 effectors. **(B)** Subcellular localization of three representative effectors in *N. benthamiana* protoplasts: cytoplasm and plasma membrane (PvAVH1), the nucleus and cytoplasm (PvAVH54804), and nuclear specific (PvAVH53). Effectors-GFP fusion proteins and GFP were transiently expressed in *N. benthamiana* protoplasts following polyethylene glycol (PEG)-mediated transformation of the protoplasts. Photographs were taken 20–24 h after transformation. Scale bars = 25 μm. **(C)** Four sub-nuclear localization patterns observed for RxLR proteins (PvAVH71, 35, 133, and 67). Photographs were taken 72 h after infiltration. Scale bars = 5 μm.

**FIGURE 5:**
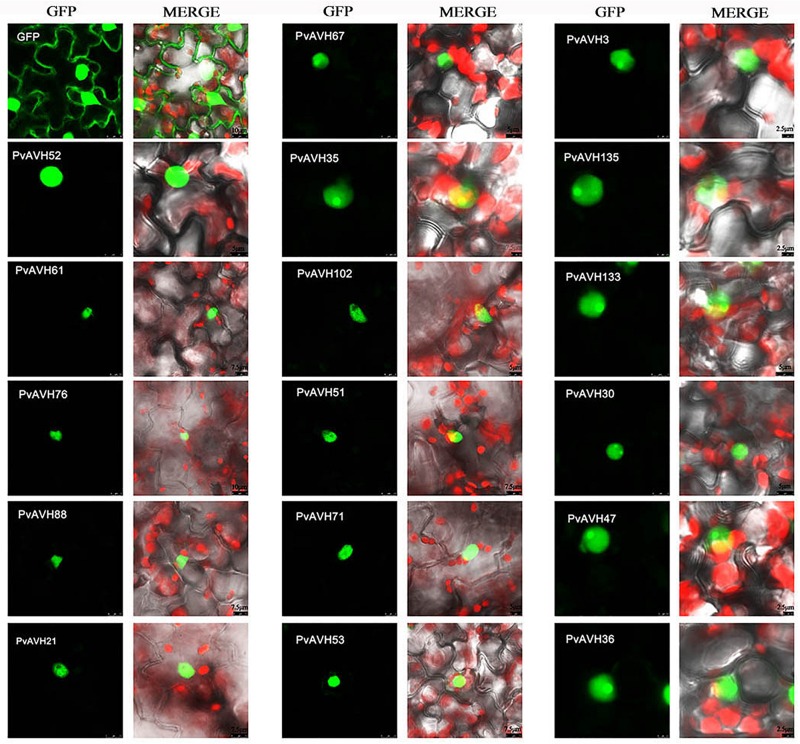
Nuclear localization of RxLRs in *Vitis vinifera.* Sub-nuclear localization of 17 RxLRs. Effectors-GFP fusion proteins were expressed in *V. vinifera* leaves using agroinfiltration. The accumulations of fluorescent protein-tagged RxLRs were captured by confocal microscopy at 72 h post-infiltration. The fluorescent of effectors-GFP fusing proteins was showed on the left panels (green channel) and the merged layer on the right panels. Scale bars = 5–10 μm.

**FIGURE 6:**
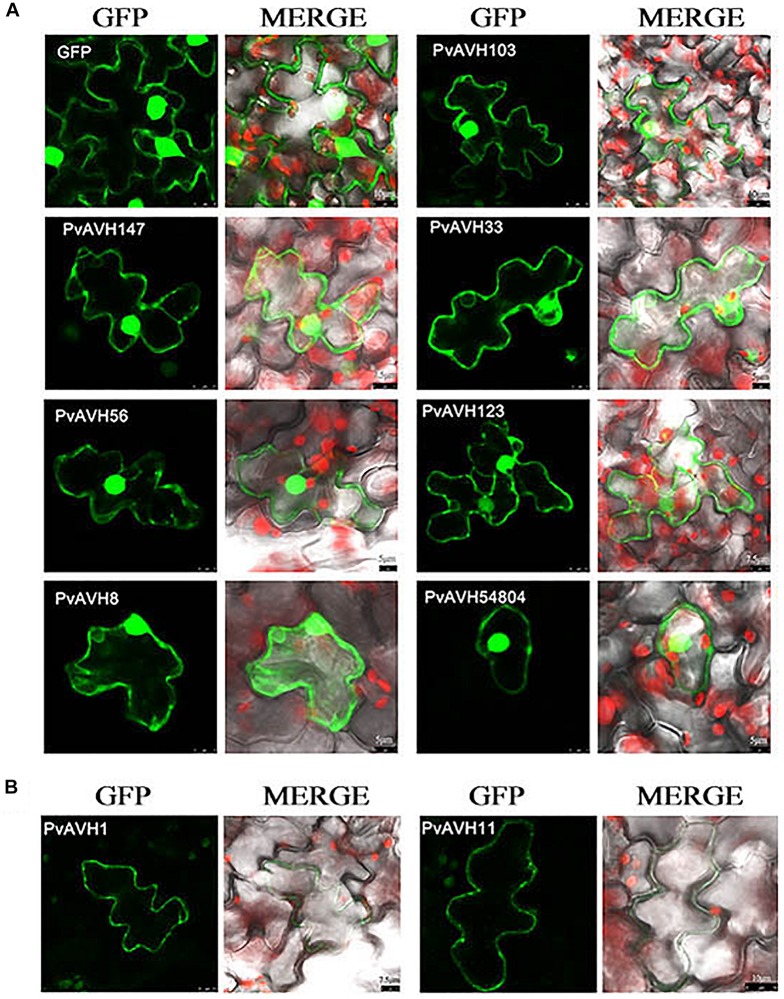
Localization of RxLRs to the nuclear and cytosolic, cytoplasm and plasma membrane in *Vitis vinifera.* **(A,B)** The nuclear and cytosolic, cytoplasm and plasma membrane localization of 9 RxLRs. GFP-effectors fusion protein were expressed in *V. vinifera* leaves using agroinfiltration. The accumulations of fluorescent protein-tagged RxLRs were captured by confocal microscopy at 72 h post-infiltration. The fluorescent of effectors-GFP fusing proteins was showed on the left panels (green channel) and the merged layer on the right panels. Scale bars = 5–10 μm.

To further analyze the function of the 26 putative *P. viticola* RxLR effectors, the ORFs were ligated into the *pCAMBIA2300* vector as fusions to GFP sequence. The plasmids were introduced into epidermal cells of *N. benthamiana* leaves through infiltration and agrobacterium-mediate transformation, and subcellular localization was visualized by confocal microscopy 3 days later (Table 2). Of the 26, 17 (65%) showed fluorescence concentrated in the nucleus, while 7 (27%) showed fluorescence in both the nucleus and cytoplasm. Two (8%) showed fluorescence in the cytoplasm and plasma membrane (Supplementary Figures S2–S4 and Figures 4A,B). Most of the 24 that were localized to the nucleus exhibited a classical nuclear localization signal (NLS) (Table 3). For each putative RxLR effector, the subcellular localizations of 26 effectors in *N. benthamiana* cells were the same as observed in *V. vinifera* (Figures 5, 6 and Supplementary Figures S5–S7).

**TABLE 3:** Canonical nuclear localization signalprediction of effectors.

**Effectors**	**Prediction results**	
	**Monopartite NLS**	**Bipartite NLS**
PvAVH51	LNNKRKRIDS	
PvAVH52	LNSKRKRIDP	
PvAVH53	LNNKRKRIDS	
PvAVH71	LHSKRKRIDP/RNSKRQRIN	
PvAVH102	LISKRKRIDP	
PvAVH67		QVKTHHLSHQVVKT PDKMNTKASSRRFLL
PvAVH135	KNKKRQRID/QKLKRLKTL	RKAKNNDKNKRSDE VKPGNKKRQRIE
PvAVH47	DMIRRKRQRID	EKNLKEAYSVKLLIMY ELFYDFCHGNKKLVG
PvAVH3	RRRTKRPRAM/VLAKRRRTKR	
PvAVH76	IVHRPKKMRLS	QGLKRWRLMYRDFF
PvAVH21	KKKLRTK	
PvAVH133	KKSKRQRIE/NIKKRQRID	
PvAVH30		RPGKQHTDLSPYDL QTPVPEKNYFQHIMSND
PvAVH88		TILKAHFNEEELLGIA EEAEKVDSTKSIA
PvAVH61	KSRKRKSST	ENLSPKSWPNNWIL QPFHYNPSRYPRHKMLQ
PvAVH35	RSNKRQRIV/GQVKRKRPNR	
PvAVH36	NNRKRNRSD	KMLPKRVLPGSRDL KNKWAVHAGGEDRMLNRI

Interestingly, for those 17 effectors that localized to the nucleus (Supplementary Table S3), we observed four, distinct subnuclear localization patterns (Figure 4C): (1) PvAVH53, 52, 51, 102, and 71 were distributed in the nucleoplasm; (2) PvAVH35 showed clear localization to the nucleoplasm and nucleolar cap-like structures; (3) PvAVH133, 76, and 21 showed clear accumulation in the nucleolus but also in foci within the nucleoplasm.; (4) PvAVH67, 135, 47, 3, 30, and 36 were concentrated in the nucleolus.

### Analysis of Expression of *P. viticola* Effector Genes During Infection of *V. vinifera*

**FIGURE 7:**
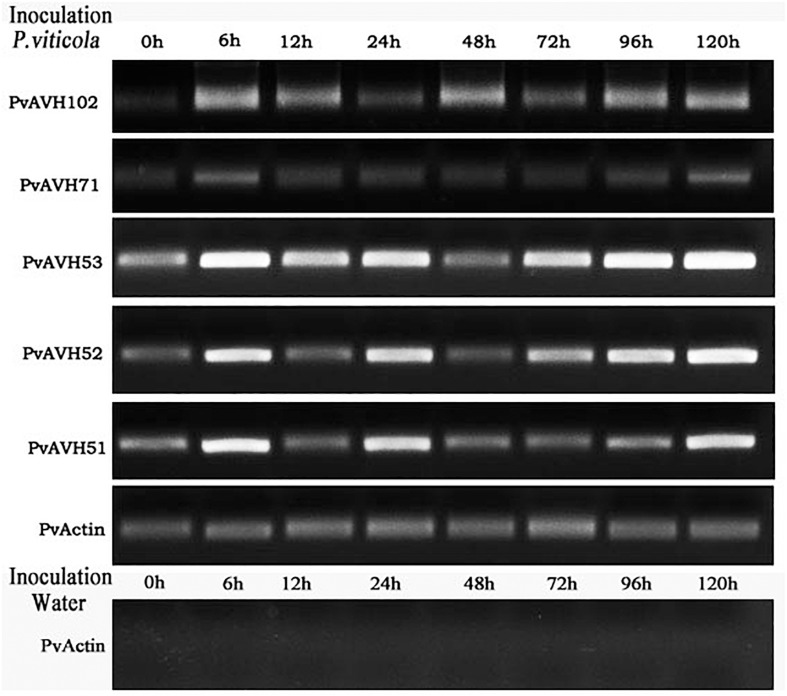
Expression of selected effectors in infected “Pinot Noir” grapevine. Expression level of five effectors (PvAVH51, 52, 53, 71, and 102) was monitored by RT-PCR at 0 h, 6 h, 12 h, 24 h, 48 h, 72 h, 96 h, and 120 h after *Plasmopara viticola* inoculation and water (as negative control). *PvActin* gene was used as an internal reference.

PvAVH51, 53, 71,102, and 52 have a close phylogenetic relationship, are located on the same chromosome, and exhibit the same subcellular localization. Three of these could induce plant cell death, while the remaining two suppressed INF1- and BAX-triggered cell death. To better understand the function of these genes in the host-pathogen interaction, we evaluated their temporal expression patterns during infection of *V. vinifera* (Figure 7). A *PvActin* gene was chosen to monitor the pathogen growth and normalize the measurement of the expression level of the effector genes. All five effector genes increased in expression by 6 hours post-inoculation (hpi) and then decreased by 12 hpi. PvAVH71 and PvAVH102 were not expressed or had a low expression level that could not be detected at 0 hpi. The relative expression of PvAVH53 was the highest at all time points, while that of PvAVH71 was the lowest. PvAVH51, PvAVH52, PvAVH71, and PvAVH53 showed a similar expression pattern that showed a low expression level at 48 hpi, and kept rising from 48 to 120 hpi. However, PvAVH102 showed a relatively high expression level at 48 hpi, and kept rising from 72 to 120 hpi. This apparent phenomenon in transcriptional response to infection suggests that each gene may be regulated to facilitate entry of the pathogen.

## Discussion

To date, knowledge of the pathology of *P. viticola* has been based on information from studies of *P. infestans*, which is a model for oomycete pathology (Bozkurt et al., 2012; King et al., 2014). RxLR-type effectors are the largest category and the most studied host-translocated proteins in oomycete. We identified effector-like proteins from *P. viticola* based on genomic and RNA-based sequence. We further identified 26 RxLR effectors for functional analysis. These sequences that blasted against the *P. viticola* the published genome “INRA-PV221” showed a stronger homology relationship. Phylogenetic analysis revealed that PvAVH51, 53, 71, 102, and 52 are closely related. Amino acid sequence alignment of these five proteins showed strong homology (up to 87.66% sequence similarity), and bioinformatics analysis revealed that the genes were located on the same chromosome. These five proteins also exhibited identical sub-nuclear localization. However, these putative effectors displayed distinct functions when expressed in *N. benthamiana* and distinct expression patterns at different stages of infection of *V. vinifera*. The presence of multiple, nearly identical gene copies has previously been reported in oomycete genomes (Mestre et al., 2016; Yin et al., 2017), and are thought to result from gene duplication during evolution, potentially facilitating infection of the host.

Characterization of the subcellular localizations of*P. viticola* effectors will be critical for understanding theplant pathogenesis. *P. viticola* is an obligate biotrophicoomycete and therefore is unable to survive *in vitro*. Transient transformation of grapevine leaves is technically challenging and inefficient. Therefore, we used transient expression in the nonhost *N. benthamiana* to study the biological function and subcellular localization of the *P. viticola* effectors. This approach has been successfully applied for many bacterial and *P. infestans* effectors (Wang et al., 2011). However, we were able to use transient expression in leaves of *V. vinifera* to examine subcellular localization, and obtained results that were identical to those from the *N. benthamiana* system. In recent years, increasing evidences reveal that the plant localization for pathogen effectors is required for their function, which probably entre the host cell compartments to modulate plant innate immune responses and subsequently contribute to pathogenicity through plant-pathogen interaction (Boch and Bonas, 2010; Caillaud et al., 2012). For example, the RxLR effector Avh241 from *Phytophthora sojae* requires plasma membrane localization to induce plant cell death (Yu et al., 2012), and PvAvh74 and PvRxLR16 from *P. viticola* induced cell death depends on nuclear localization (Xiang et al., 2016; Yin et al., 2019). The effector AVR1 from *P. infestans* active the innate immune response by resistance protein R1 depending on the nuclear colocalization, and it also can target the exocyst to disturb the vesicle trafficking for potentially regulating plant immunity by interacting with exocyst subunit Sec5 protein (Du et al., 2015a, b). These results showed that the localization of the effectors were important to study the interaction between *P. viticola* and *Vitis*. Our functional analysis of the proteins was based on their ability to induce PCD or suppress the PCD triggered by INF and BAX in *N. benthamiana*. The number of pathogen effectors that can induce cell death is relatively small (Liu et al., 2018). Among the 26 RxLR effectors studied here, we found that seven could induce cell death, and that some of these could trigger a more rapid and stronger response than the others. Although effectors may often have redundant function, several individual effectors are keys to the full virulence of pathogen. For example, AVH241 and AVH238 could induce cell death and promote infection of *P. sojae* in *N. benthamiana* (Yu et al., 2012; Yang et al., 2017, 2019). Therefore, we hypothesized that 26 effectors might regulate the plant defense responses to contribute to virulence of *P. viticola*. Suppression of plant immunity responses is considered to be the primary activity of the *P. viticola* effectors (Liu et al., 2018). To evaluate this activity, we examined their ability to suppress cell death induced by INF1 and BAX. We found that most of the effectors tested could suppress cell death by BAX, while fewer could suppress cell death induced by INF1.

To assess the subcellular localization of the effectors, we carried out transient expression experiments in *N. benthamiana* leaves and protoplasts. Recently, increasing evidence suggests that while oomycete effectors can localize to a variety of plant cell compartments, the majority localize to the nucleus. For example, 82% of RxLR effectors from *P. viticola* and 66% of RxLRs from *Hyaloperonospora arabidopsidis* tested were found to be targeted to the nucleus (Caillaud et al., 2012; Liu et al., 2018). Our results are consistent with these reports, as 68% of the *P. viticola* effectors we studied were targeted to the nucleus and 24% were targeted to the nucleus and cytoplasm. Furthermore, we found that *P. viticola* effectors showed multiple sub-nuclear localizations. The majority mainly localized to nucleolus with additional weak localization to the nucleoplasm, but PvAVH35 strongly localized nucleolar cap-like structures. Others, such as PvAVH71, localized to the nucleoplasm. The significance of localization to discrete subcellular compartments is unknown. Previous studies revealed that effectors that localize to the nucleus may participate in reprogramming transcriptional mechanisms to suppress the plant innate immune response. For example, the *P. infestans* effector AVR2 suppresses plant innate immunity by up-regulating a brassinosteroid-responsive bHLH transcription factor (Boch and Bonas, 2010; Turnbull et al., 2017).

The nearly identical copies observed for some of the genes may be induced by LTR transposons, DNA transposons or other elements (Qutob et al., 2009; Mestre et al., 2016). PvAVH51, 53, 71, 102, and 52 are very closely related. These effectors (namely 51, 53, and 71) caused necrosis in *Nicotiana* leaves, but no cell death was observed in grape (*V. vinifera*) leaves. This phenomenon has been reported in three *P. viticola* RxLR effectors, PVITv1021061, PVITv1008294, and PVITv1008311 (Brilli et al., 2018). Meanwhile, a similar phenomenon was found that a *P. infestans* effector PITG_22798 induced cell death in tobacco but not in potato species (Wang et al., 2017). The cause of this result may be that these effectors can not be identified by the grapevine to trigger a immune response like cell death. Expression of these five effectors could be detected in infected grapevine leaves using RT-PCR, and were induced at an early stage of infection. All five effectors genes increased sharply at 6 hpi, but decreased at 12 hpi immediately. This similar expression patterns of RxLR effectors have been founded in *H. arabidopsidis* and *P. sojae*, namely “immediate-early, low” (Wang et al., 2011; Anderson et al., 2012). As previously reported, *P. infestans* haustoria appeared in the infected tissue at 3 hpi, and were abundant at 12 hpi (Wang et al., 2011). *P. viticola* encysted zoospores were observed on the stoma at 12 hpi (Yin et al., 2017). Moreover, PvRxLR18 and PvRxLR28 showed increased expression at 6 hpi, and this phenomenon was obvious in the highly virulent strains (Gomez-Zeledon and Spring, 2018). While PvAVH51, 53, 71, and PvAVH52 showed decreased expression levels after 24 h and then an increasing trend of up to 120 h. These similar expression patterns also have been reported for the effectors of *P. viticola* like PvRxLR28 and PvRxLR5 (Xiang et al., 2016). The *P. viticola* encysted zoospores were observed on the stoma at 12 hpi and the hyphae could be observed in spongy mesophyll at hpi (Yin et al., 2017). During the time (24–72 hpi), *P. viticola* may be busy in producing haustoria to obtain nutrients for growth and development at 48 hpi. Meanwhile, with the numbers of *P. viticola* increasing, the new infection will start, and the expression of these effectors will increase. Therefore, strong expression of effector genes early (prior to 12 h) during infection likely facilitates entry of the pathogen.

In summary, our results provide insights into the biological activities and subcellular localization of 26 *P. viticola* RxLR effectors. *P. viticola* RxLR effectors showed similar localization in *N. benthamiana* and *V. vinifera.* The majority of the studied effectors localized to the nucleus, and may function to suppress plant innate immunity responses. Further study of the pathogenic molecular mechanisms of *P. viticola* will provide a theoretical foundation for understanding downy mildew disease in grapevine. This will enable the development of innovative approaches to control the disease as well as new, downy mildew-tolerant grapevine cultivars. To date, our understanding of how *P. viticola* effectors modulate plant immunity is still limited. Therefore, we will focus on the interaction between plant and pathogen, and the mechanism involved in the fight against infection.

## Data Availability Statement

The datasets generated for this study can be found in the all datasets for this study are included in the Supplementary Material.

## Author Contributions

TC and YX designed the research, and MD cloned all of the putative effectors genes and constructed the vectors in this study. TC and RL performed the analyses of subcellular localization and suppression. MYL and MJL prepared the plant materials. TC drafted the manuscript. XY, GL, and YW modified the language. All authors have read and approved the final manuscript.

## Conflict of Interest

The authors declare that the research was conducted in the absence of any commercial or financial relationships that could be construed as a potential conflict of interest.

## Footnotes

1 http://www.cbs.dtu.dk/services/SignalP/

2 http://nls-mapper.iab.keio.ac.jp/cgi-bin/NLS_Mapper_form.cgi#opennewwindow
