# Supplementary material for: Insight Into Function and Subcellular Localization of Plasmopara viticola Putative RxLR Effectors
Source: Front Microbiol. 2020 Apr 21;11:692. doi: 10.3389/fmicb.2020.00692 (PMC7186587; doi:10.3389/fmicb.2020.00692)
Supplement: Supplementary file 1 [file Data_Sheet_1.docx]

Supplementary Material

Insight into function and subcellular localization of *Plasmopara viticola* putative RxLR effectors

# Supplementary Figures 1

##
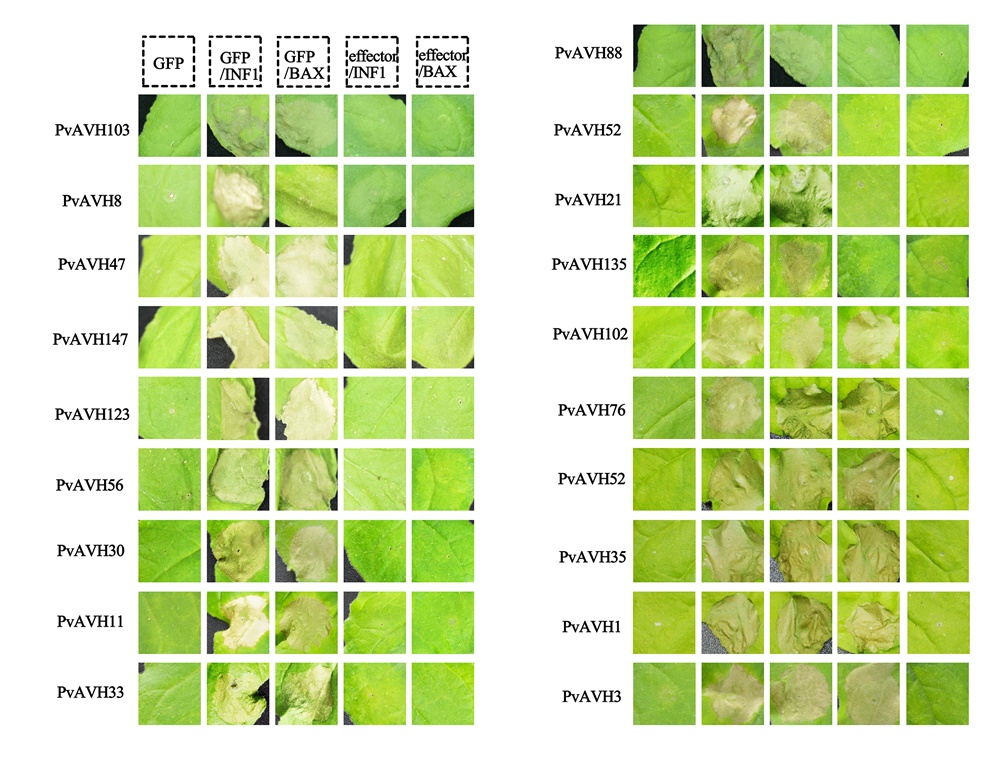


**Fig. S1 Assay for suppression of BAX and INF1-triggered cell death in *N.benthamiana* by RxLRs.**

(A, B) Effector -GFP fusion proteins and GFP (as negative control) were expressed 24 h earlier than PVX-INF1 and PVX-BAX *(*as positive control*)* in *N.benthamiana* leaves using agroinfiltration. Photographs were taken 5d post infiltration.

# Supplementary Figures 2


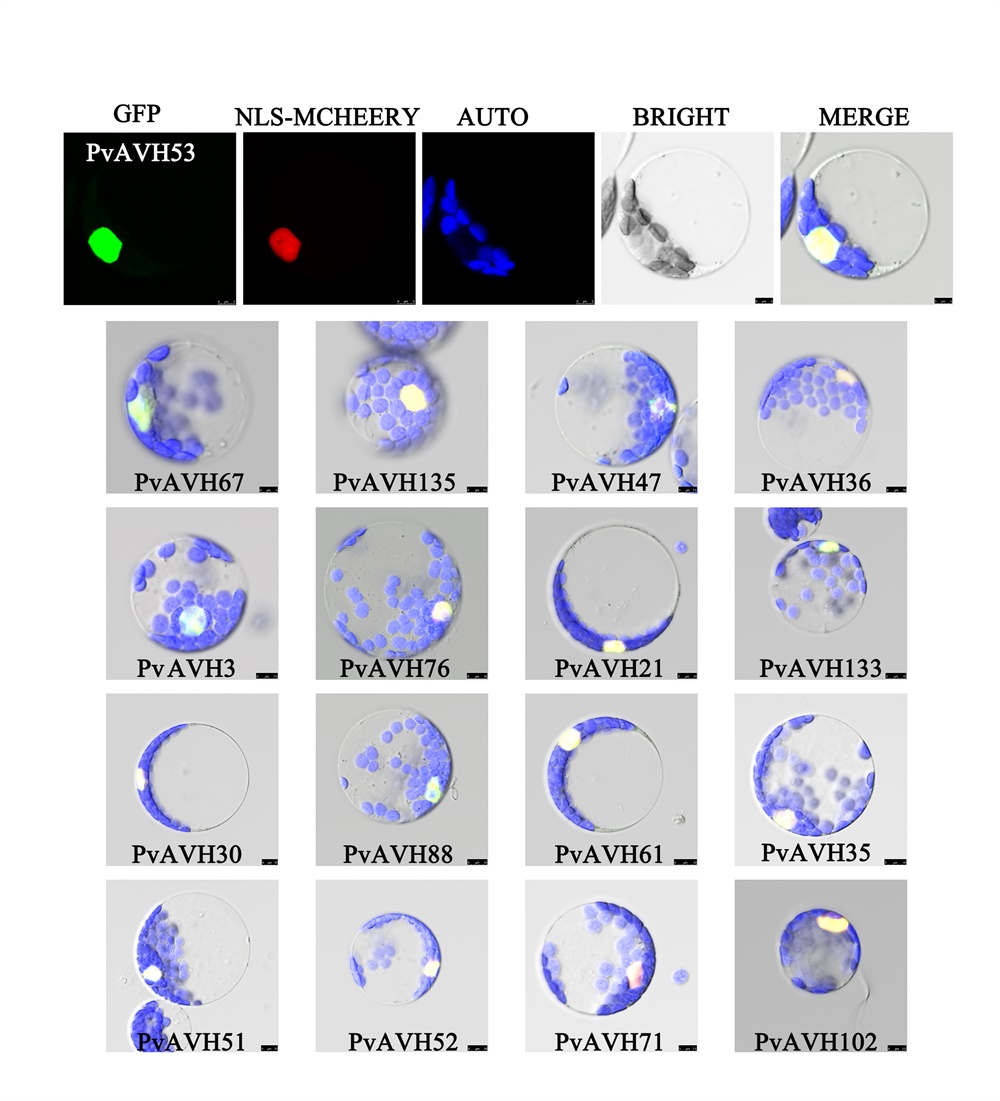


**Fig. S2 Nuclear localization of RxLRs in *N. benthamiana* protoplasts**

The 17 nuclear effectors constructs were introduced into *N. benthamiana* protoplasts through polyethylene glycol (PEG)-mediated transformation, and protoplasts were imaged 20-24 h after transformation. NLS-mCherry which was considered as nuclear-localized marker were co-expressed in *Nicotiana* protoplasts. Scale bars = 10 μm

# Supplementary Figures 3


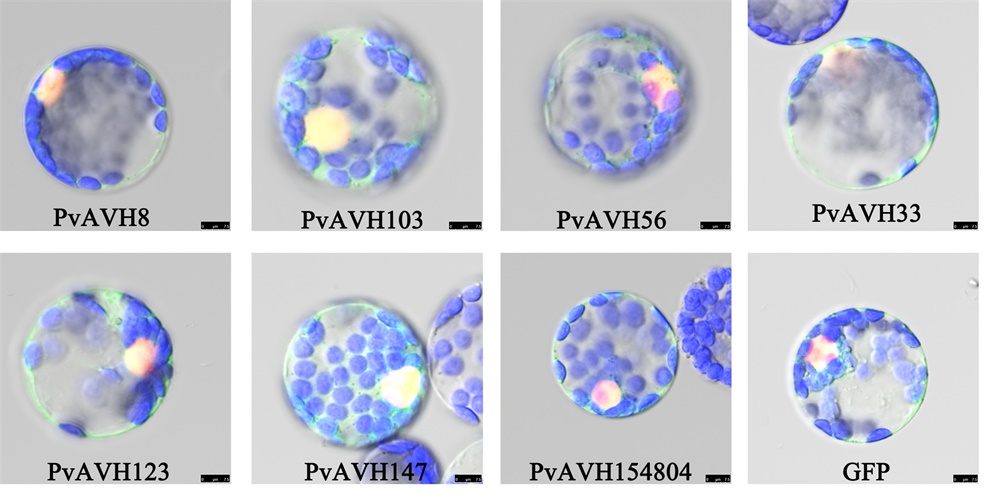


**Fig. S3 Nuclear and cytoplasmic of RxLRs in *N. benthamiana* protoplasts**

The 7 nuclear and cytoplasmic localizations of RxLR constructs were introduced into *N. benthamiana* protoplasts through polyethylene glycol (PEG)-mediated transformation, and protoplasts were imaged 20-24 h after transformation. Scale bars = 7.5μm.

# Supplementary Figures 4


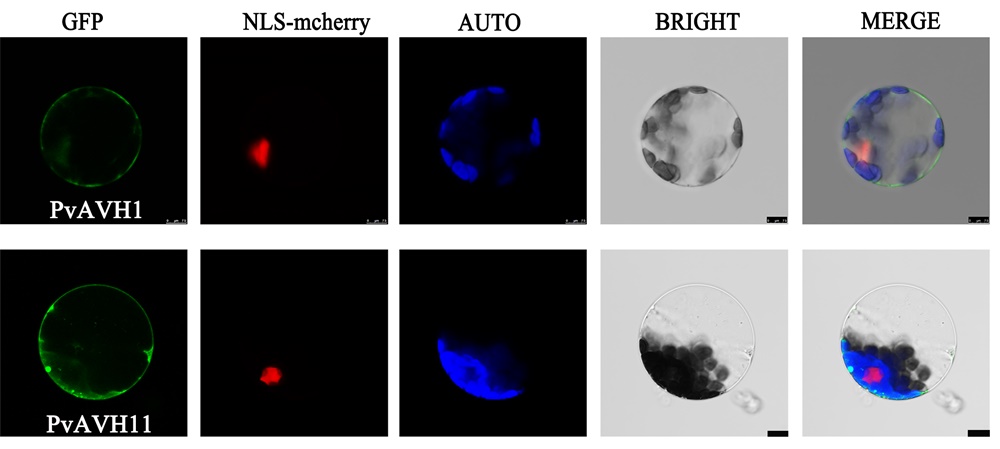


**Fig. S4 Nuclear and cytoplasmic of RxLRs in *N. benthamiana* protoplasts**

The 2 cytoplasmic localization of RxLRs. GFP-effectors fusion protein were expressed in *N. benthamiana* protoplasts through polyethylene glycol (PEG)-mediated transformation, and protoplasts were imaged 20-24 h after transformation. NLS-mCherry which was considered as nuclear-localized marker were co-expressed in *Nicotiana* protoplasts. Scale bars = 7.5μm.

# Supplementary Figures 5


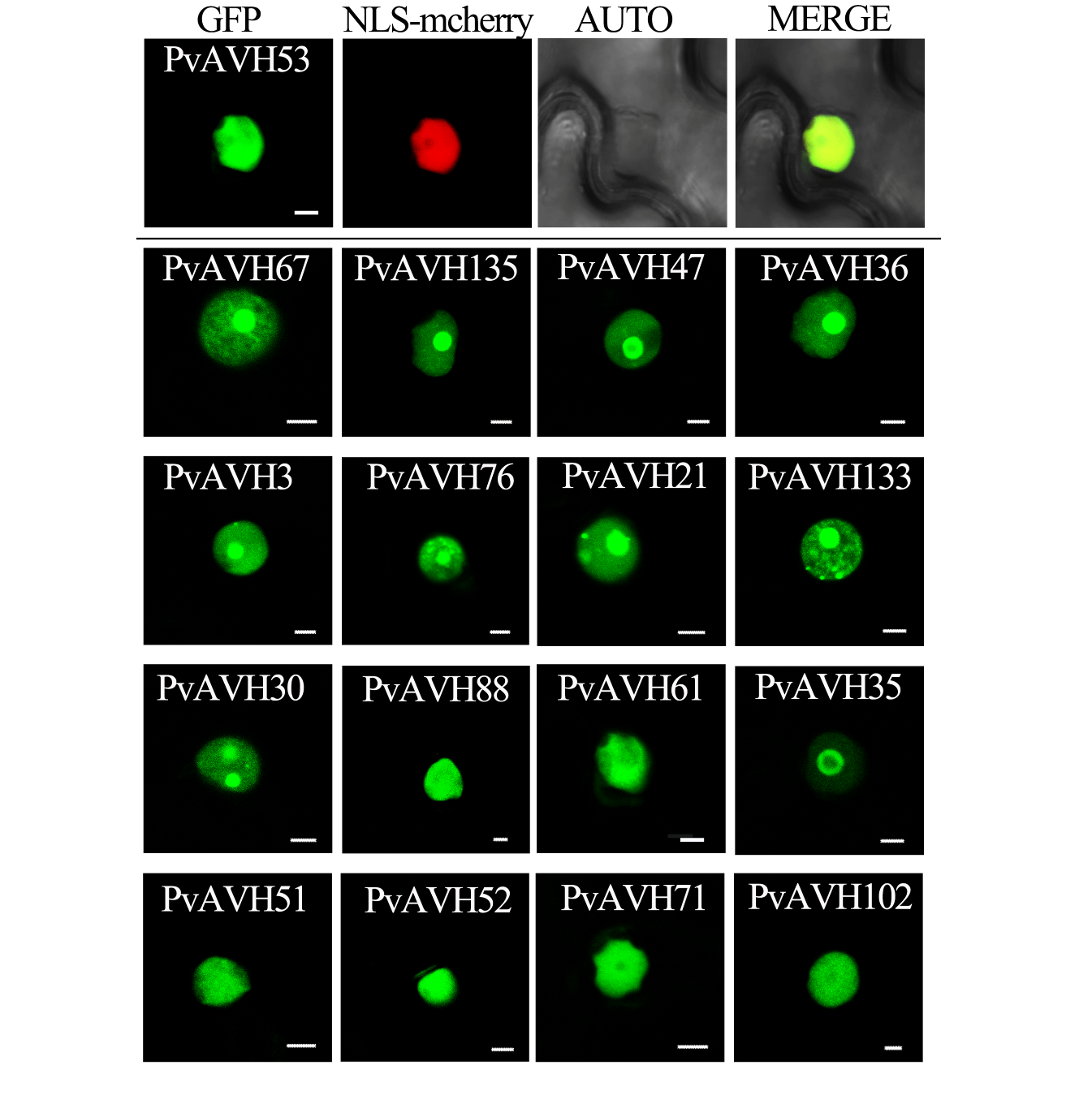


**Fig. S5 Nuclear localization of RxLRs in *N. benthamiana***

The 17 sub-nuclear localization of RxLRs. Effector-GFP fusion proteins were transiently agroinfiltrated in *N. benthamiana* leaves. NLS-mCherry which was considered as nuclear-localized marker were co-expressed in *Nicotiana* leaves .The fluorescent protein-tagged effectors were captured by confocal microscopy 72 hpi. Scale bars = 5 μm

# Supplementary Figures 6


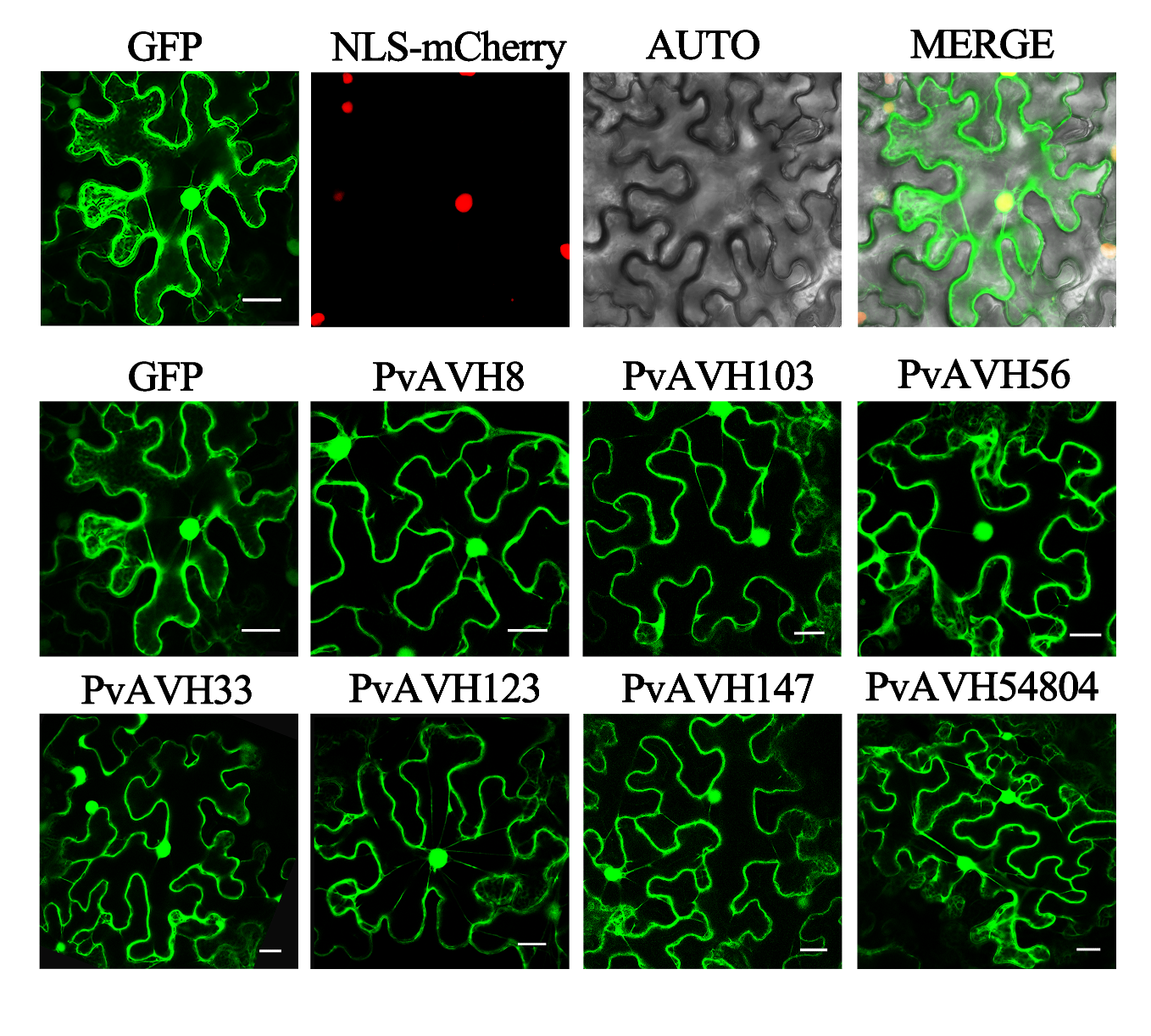


**Fig. S6 Nuclear and cytoplasmic localization of RxLRs in *N. benthamiana***

The 7 nuclear and cytoplasmic localization of RxLRs. Effector-GFP fusion proteins or GFP were agroinfiltrated into *N.benthamiana* leaves. NLS-mCherry which was considered as nuclear-localized marker were co-expressed in *Nicotiana* leaves. The fluorescent protein-tagged effectors was captured by confocal microscopy 72 hpi. Scale bars = 20 μm

# Supplementary Figures 7

**
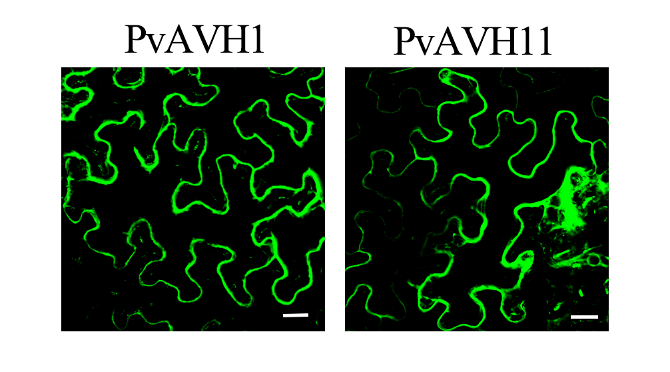
**

**Fig. S7 Localization of RxLRs to the plasma membrane and cytoplasm in *N. benthamiana***

The 2 cytoplasmic localization of RxLRs. Effectors-GFP fusion protein were agroinfiltrated in *N.benthamiana* leaves. The fluorescent protein-tagged effectors was captured by confocal microscopy 72 hpi. Scale bars = 20 μm

# Supplementary Figures 8

**
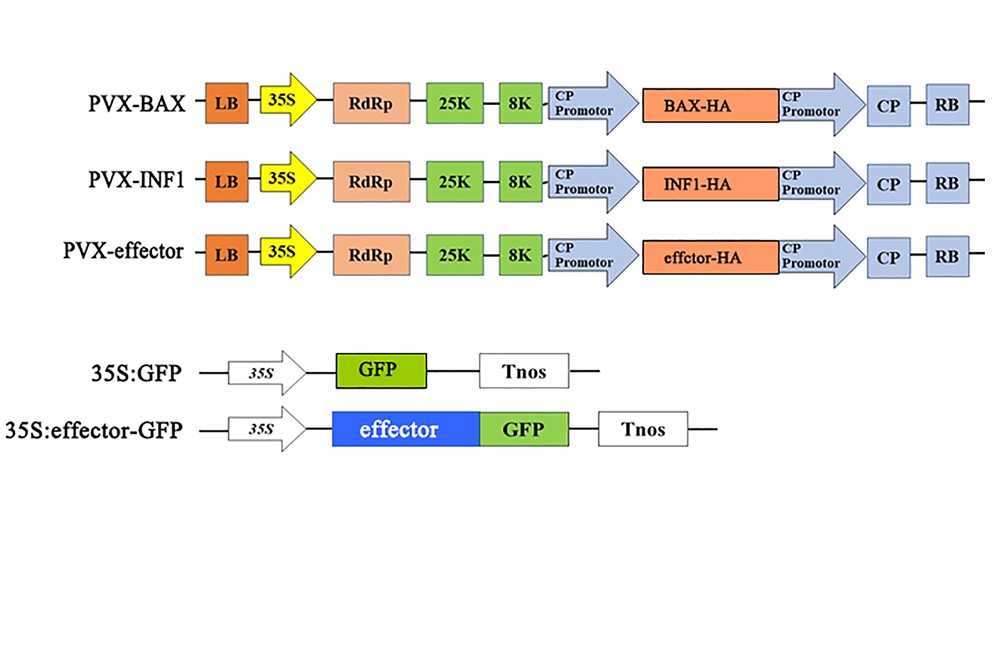
**

**Fig. S8 The schematic diagrams of the constructs used for experiments**

**Table S1 List of primers used for plasmid construction**

| **genes** | **Primers for Pcambia2300** |
| --- | --- |
| AVH52-kpn1-f | Ggtacc ATG ACT GCA GGC TTT GAC CAG GCC |
| AVH52-xba1-r | TCTAGA ATG CTC AAA AAT CGA TTG ATG GGA GTT GAC |
| AVH56-kpn1-f | Ggtacc ATG TTC AAA CAT AAT GAG CCT CTT CAT GCA TC |
| AVH56-bamh1-r | Aggatcc AAG CTT CAA GAT CCA ACT TGC AGG AC |
| AVH67-sac1-f | gagctc ATG AAG TCT AAC CAA GTA AAA ACT CAT CAT CTG T |
| AVH67-bamh1-r | ggatcc AGT ATG GGA AGC GGC GAG AG |
| AVH61-sac1-f | gagctc ATG GAG TTT AAC CAA GAC GAG TGT CAA CAA |
| AVH61-bamh1-r | ggatcc TCG AGA CTG TAA CAT TTT ATG TCT TGG G |
| AVH35-sac1-f | gagctc ATG GCG AAG TGT GAT CAA GAT GAG CCT |
| AVH35-bamh1-r | ggatcc ATC ATG AGG AGG CAG GAC TTG AC |
| AVH33-sac1-f | gagctc ATG TTT GAC CAG GAT GAA CCT CAA CAT G |
| AVH33-bamh1-r | ggatcc GTG ATT CTT GTC GCC ACT GCT TG |
| AVH103-sac1-f | gagctc ATG AGC AGC TCT GCG ATT CTC GAT C |
| AVH103-bamh1-r | ggatcc CCT GCG TTG GTA AGG TGG TAC AA |
| AVH30-sac1-f | gagctc ATG GAG TCT TAC CAA ATT GAT CCT CGA CAA |
| AVH30-bamh1-r | ggatcc AAA GCG TCG AGG TTG CTT TCC TC |
| AVH3-kpn1-f | Ggtacc ATG GAG TTT GTC CAA GAT GAT CTT CAA CAA AC |
| AVH3-xba1-r | TCTAGA ATC AGG AAG TGA AAC TTG ATG ATT CTT ATT GC |
| AVH47-kpn1-f | ATG CGT GGC ACA ATT TAC GTG G |
| AVH47-xba1-r | TTA AGT TGT AAC CTT CAG GAT CGA AT |
| AVH102-kpn1-f | Ggtacc ATG ACT GCA GGC TTT GAC CAG GCC |
| AVH102-xba1-r | TCTAGA ATG CTC AAA TAT CGA TTG ATG GGA TCT GAC |
| AVH71-kpn1-f | Ggtacc ATG AGT GCA GGA TTT GAC CAG GCC |
| AVH71-xba1-r | TCTAGA ATG CTC AAA AAT CGA TTG CTG GGA TTT G |
| AVH51-sac1-f | gagctc ATG GAA TTT GAC GAG GCC GAG CCT C |
| AVH51-bamh1-r | ggatcc ATG ATC AAA AAT CGA TTG ATG GGA TTT GCC |
| AVH36-kpn1-f | Ggtacc ATG GCT GAA GTT GAC CAG ACC GAG |
| AVH36-xba1-r | TCTAGA AAT TAA AAG CTC CAG GAT CCA ATG GC |
| AVH123-sac1-f | gagctc ATG GAT CAA CCA GGA CGC CAA CCA ATG T |
| AVH123-bamh1-r | ggatcc GTC ATG TAG AAT GTG GTA TGC CAC CAA C |
| AVH147-kpn1-f | Ggtacc ATG ATT AGT GTG GCA TTA GAG GAA AAT GCG AC |
| AVH147-xba1-r | TCTAGA CGA GTC ATC CTT TGG TTT AAT CTG TGG |
| AVH88-kpn1-f | Ggtacc ATG GAG GCC AAT GCG ACG CGT G |
| AVH88-xba1-r | TCTAGA AGC TGA CGA TTT AGG CAC ATT CTG C |
| AVH11-kpn1-f | Ggtacc ATG TCC ACC GAG TCG AAT GTA GTG TCT T |
| AVH11-xba1-r | TCTAGA CAC GGA CCG AAG GTT TTC AGT GC |
| AVH133-sac1-f | gagctc ATG GAG CCT GAC CAG GCC GAG |
| AVH133-bamh1-r | ggatcc AGT TGA AAG CTT GAG GAC CCA ATC AC |
| AVH135-sac1-f | gagctc ATG GAC CAG GCC GAG CCT CAC |
| AVH135-bamh1-r | ggatcc AGT CGA TAC CTT CAG GAT CGA ATC AC |
| AVH53-sac1-f | gagctc ATG GAA TTT GAC CAG GCC GAG CCT C |
| AVH53-bamh1-r | ggatcc TTG CTC AAA AAT CGA TTG ATG GGA TTT GAT TTT C |
| AVH1-kpn1-f | Ggtacc ATG GTC CCA CGT GGC TCT CTG AG |
| AVH1-xba1-r | TCTAGA CGG ATT CAG GTA TTT CGG AGG ATT GAT |
| AVH8-kpn1-f | Ggtacc ATG ACG AAC GCC GTG ACG GCG CAT |
| AVH8-xba1-r | TCTAGA ATG AGC GAC AAC AAC AGG TGC ATG AAC |
| AVH76-kpn1-f | Ggtacc ATG GCC GCC GAG GAA CAA CAA CAA AC |
| AVH76-xba1-r | TCTAGA AGA AGT TTC TGC GTG ATG GTT TCC CC |
| AVH21-sac1-f | Gagctc ATG GAT CGA TCG GAA TAC CGA CAA AGG TC |
| AVH21-bamh1-r | ggatcc GAG CCG CTG AGT AAT TGA CTC CTT G |
| AVH54804-Sac1-f | gggGAGCTC-ATG TCT TCC GTG AAT AGA ACA GAG TTG ATT |
| AVH54804-BamH1-r | gggGGATCC-ATT TAC ATT GAC ATC GCT AGC TCG T |
|  |  |
| **genes** | **Primers for PVX** |
| PvAVH52-Sma1-f | CCCCGGGG ATG ACT GCA GGC TTT GAC CAG GCC |
| PvAVH52-Not1-r | TTGCGGCCGCAA ATG CTC AAA AAT CGA TTG ATG GGA GTT GAC |
| PvAVH56-Sma1-f | CCCCGGGG ATG TTC AAA CAT AAT GAG CCT CTT CAT GCA TC |
| PvAVH56-Not1-r | TTGCGGCCGCAA AAG CTT CAA GAT CCA ACT TGC AGG AC |
| PvAVH67-Sma1-f | CCCCGGGG ATG AAG TCT AAC CAA GTA AAA ACT CAT CAT CTG T |
| PvAVH67-Not1-r | TTGCGGCCGCAA AGT ATG GGA AGC GGC GAG AG |
| PvAVH61-Sma1-f | gagctc ATG GAG TTT AAC CAA GAC GAG TGT CAA CAA |
| PvAVH61-Not1-r | TTGCGGCCGCAA TCG AGA CTG TAA CAT TTT ATG TCT TGG G |
| PvAVH35-Sma1-f | gagctc ATG GCG AAG TGT GAT CAA GAT GAG CCT |
| PvAVH35-Not1-r | TTGCGGCCGCAA ATC ATG AGG AGG CAG GAC TTG AC |
| PvAVH33-Sma1-f | gagctc ATG TTT GAC CAG GAT GAA CCT CAA CAT G |
| PvAVH33-Not1-r | TTGCGGCCGCAA GTG ATT CTT GTC GCC ACT GCT TG |
| PvAVH103-Sma1-f | gagctc ATG AGC AGC TCT GCG ATT CTC GAT C |
| PvAVH103-Not1-r | TTGCGGCCGCAA CCT GCG TTG GTA AGG TGG TAC AA |
| PvAVH30-Sma1-f | gagctc ATG GAG TCT TAC CAA ATT GAT CCT CGA CAA |
| PvAVH30-Not1-r | TTGCGGCCGCAA AAA GCG TCG AGG TTG CTT TCC TC |
| PvAVH3-Sma1-f | CCCCGGGG ATG GAG TTT GTC CAA GAT GAT CTT CAA CAA AC |
| PvAVH3-Not1-r | TTGCGGCCGCAA ATC AGG AAG TGA AAC TTG ATG ATT CTT ATT GC |
| PvAVH47-Sma1-f | ATG CGT GGC ACA ATT TAC GTG G |
| PvAVH47-Not1-r | TTA AGT TGT AAC CTT CAG GAT CGA AT |
| PvAVH102-Sma1-f | CCCCGGGG ATG ACT GCA GGC TTT GAC CAG GCC |
| PvAVH102-Not1-r | TTGCGGCCGCAA ATG CTC AAA TAT CGA TTG ATG GGA TCT GAC |
| PvAVH71-Sma1-f | CCCCGGGG ATG AGT GCA GGA TTT GAC CAG GCC |
| PvAVH71-Not1-r | TTGCGGCCGCAA ATG CTC AAA AAT CGA TTG CTG GGA TTT G |
| PvAVH51-Sma1-f | gagctc ATG GAA TTT GAC GAG GCC GAG CCT C |
| PvAVH51-Not1-r | TTGCGGCCGCAA ATG ATC AAA AAT CGA TTG ATG GGA TTT GCC |
| PvAVH36-Sma1-f | CCCCGGGG ATG GCT GAA GTT GAC CAG ACC GAG |
| PvAVH36-Not1-r | TTGCGGCCGCAA AAT TAA AAG CTC CAG GAT CCA ATG GC |
| PvAVH123-Sma1-f | gagctc ATG GAT CAA CCA GGA CGC CAA CCA ATG T |
| PvAVH123-Not1-r | TTGCGGCCGCAA GTC ATG TAG AAT GTG GTA TGC CAC CAA C |
| PvAVH147-Sma1-f | CCCCGGGG ATG ATT AGT GTG GCA TTA GAG GAA AAT GCG AC |
| PvAVH147-Not1-r | TTGCGGCCGCAA CGA GTC ATC CTT TGG TTT AAT CTG TGG |
| PvAVH88-Sma1-f | CCCCGGGG ATG GAG GCC AAT GCG ACG CGT G |
| PvAVH88-Not1-r | TTGCGGCCGCAA AGC TGA CGA TTT AGG CAC ATT CTG C |
| PvAVH11-Sma1-f | CCCCGGGG ATG TCC ACC GAG TCG AAT GTA GTG TCT T |
| PvAVH11-Not1-r | TTGCGGCCGCAA CAC GGA CCG AAG GTT TTC AGT GC |
| PvAVH133-Sma1-f | gagctc ATG GAG CCT GAC CAG GCC GAG |
| PvAVH133-Not1-r | TTGCGGCCGCAA AGT TGA AAG CTT GAG GAC CCA ATC AC |
| PvAVH135-Sma1-f | gagctc ATG GAC CAG GCC GAG CCT CAC |
| PvAVH135-Not1-r | TTGCGGCCGCAA AGT CGA TAC CTT CAG GAT CGA ATC AC |
| PvAVH53-Sma1-f | gagctc ATG GAA TTT GAC CAG GCC GAG CCT C |
| PvAVH53-Not1-r | TTGCGGCCGCAA TTG CTC AAA AAT CGA TTG ATG GGA TTT GAT TTT C |
| PvAVH1-Sma1-f | CCCCGGGG ATG GTC CCA CGT GGC TCT CTG AG |
| PvAVH1-Not1-r | TTGCGGCCGCAA CGG ATT CAG GTA TTT CGG AGG ATT GAT |
| PvAVH8-Sma1-f | CCCCGGGG ATG ACG AAC GCC GTG ACG GCG CAT |
| PvAVH8-Not1-r | TTGCGGCCGCAA ATG AGC GAC AAC AAC AGG TGC ATG AAC |
| PvAVH76-Sma1-f | CCCCGGGG ATG GCC GCC GAG GAA CAA CAA CAA AC |
| PvAVH76-Not1-r | TTGCGGCCGCAA AGA AGT TTC TGC GTG ATG GTT TCC CC |
| PvAVH21-Sma1-f | gagctc ATG GAT CGA TCG GAA TAC CGA CAA AGG TC |
| PvAVH21-Not1-r | TTGCGGCCGCAA GAG CCG CTG AGT AAT TGA CTC CTT G |
| PvAVH54804-Sma1-f | GAGCTC-ATG TCT TCC GTG AAT AGA ACA GAG TTG ATT |
| PvAVH54804-Not1-r | TTGCGGCCGCAA-ATT TAC ATT GAC ATC GCT AGC TCG T |

**Table S2 List of primer pairs were designed specially for RT-PCR**

| **genes** | **primer** |
| --- | --- |
| 51-F | GCA GAT AAT GTG ATT GCG AAT GCT GAA |
| 51-R | CATTCGGCTTAGGCGTATGCAG |
| 52-F | GCT GAT CGC TCA GAA GAC GG |
| 52-R | CC TTA TCA TCT ACC ACG ACT TCA AAA T |
| 53-F | GAT TCA AGC AAT TGA AAC CTG TGC TAG |
| 53-R | GT CGG CTC TAA ATG TAA AGT AGC CAT |
| 71-F | TC GTG GTA GAT GAT AAG GTG AAA GG |
| 71-R | CG GTG TTT GGA CTC GAG TTG ACT |
| 102-F | GGGACTGGTGAGTCGGCTTT |
| 102-R | GCCCACCGCCAAGGTAGAAT |
| PvACTIN-F | CTCCAgAACgTgTACATCCg |
| PvACTIN -R | TAgCgCCCTTCTCCTCAg |
